# Flight Modes in Migrating European Bee-Eaters: Heart Rate May Indicate Low Metabolic Rate during Soaring and Gliding

**DOI:** 10.1371/journal.pone.0013956

**Published:** 2010-11-11

**Authors:** Nir Sapir, Martin Wikelski, Marshall D. McCue, Berry Pinshow, Ran Nathan

**Affiliations:** 1 Movement Ecology Laboratory, Department of Evolution, Systematics and Ecology, Alexander Silberman Institute of Life Sciences, The Hebrew University of Jerusalem, Jerusalem, Israel; 2 Max Planck Institute for Ornithology, Vogelwarte Radolfzell, Radolfzell, Germany; 3 Department of Biological Sciences, St. Mary's University, San Antonio, Texas, United States of America; 4 Mitrani Department of Desert Ecology, Jacob Blaustein Institutes for Desert Research, Ben-Gurion University of the Negev, Midreshet Ben-Gurion, Israel; 5 Department of Biology, Konstanz University, Konstanz, Germany; Roehampton University, United Kingdom

## Abstract

**Background:**

Many avian species soar and glide over land. Evidence from large birds (*m*_b_>0.9 kg) suggests that soaring-gliding is considerably cheaper in terms of energy than flapping flight, and costs about two to three times the basal metabolic rate (BMR). Yet, soaring-gliding is considered unfavorable for small birds because migration speed in small birds during soaring-gliding is believed to be lower than that of flapping flight. Nevertheless, several small bird species routinely soar and glide.

**Methodology/Principal Findings:**

To estimate the energetic cost of soaring-gliding flight in small birds, we measured heart beat frequencies of free-ranging migrating European bee-eaters (*Merops apiaster*, *m*_b_∼55 g) using radio telemetry, and established the relationship between heart beat frequency and metabolic rate (by indirect calorimetry) in the laboratory. Heart beat frequency during sustained soaring-gliding was 2.2 to 2.5 times lower than during flapping flight, but similar to, and not significantly different from, that measured in resting birds. We estimated that soaring-gliding metabolic rate of European bee-eaters is about twice their basal metabolic rate (BMR), which is similar to the value estimated in the black-browed albatross *Thalassarche* (previously *Diomedea*) *melanophrys*, *m*_b_∼4 kg). We found that soaring-gliding migration speed is not significantly different from flapping migration speed.

**Conclusions/Significance:**

We found no evidence that soaring-gliding speed is slower than flapping flight in bee-eaters, contradicting earlier estimates that implied a migration speed penalty for using soaring-gliding rather than flapping flight. Moreover, we suggest that small birds soar and glide during migration, breeding, dispersal, and other stages in their annual cycle because it may entail a low energy cost of transport. We propose that the energy cost of soaring-gliding may be proportional to BMR regardless of bird size, as theoretically deduced by earlier studies.

## Introduction

Avian species of a wide size range, from the 25 g little swift (*Apus affinis*) to the 11 kg Andean condor (*Vultur gryphus*), soar over land on convective thermals, gaining altitude that allows horizontal progress by gliding, when seeking food, mates, and breeding sites and during long distance migratory journeys [1], [2]. Based on scaling arguments of muscle power and wing dimensions, Pennycuick [3] suggested that, unlike flapping flight, soaring flight may increase with a scaling factor similar to that of basal metabolic rate (BMR). He also proposed that birds spend about 1.5 times their BMR during soaring and gliding because the energy cost of muscle tension to maintain wing posture during soaring or gliding is about half the BMR. Yet, measurements of rate of oxygen consumption (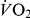) during flight in a wind-tunnel showed that for two 0.9 kg American herring gulls (*Larus argentatus smithsonianus*), metabolic rate (MR) during gliding averaged 1.9–2.4 times more than resting MR [4] and about three times their measured basal metabolic rate (BMR) [5], [6]. In wandering albatross (*Diomedea exulans*), the energy cost of soaring flight, measured using doubly labeled water (DLW), was estimated to be 2.4 times BMR [7], while measurements of heart beat frequency (*f*_H_) [8] in black-browed albatrosses (*Thalassarche* (previously *Diomedea*) *melanophrys*) estimated it to be twice that at the BMR [9]. All these measurements were made in relatively large birds (*m*_b_>0.9 kg) that were not migrating. To the best of our knowledge, the energetic cost of soaring and gliding during migration in small birds has not been studied so far.

Measurement of *f*_H_ alone (e.g., [10], [11], [12]) cannot reveal the metabolic demands of flight and other activities of free ranging animals, since *f*_H_ is only one component affecting the metabolic rate of an animal [13]. Fick's principle states that 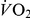 is equal to the product of the *f*_H_, stroke volume (*V*_s_, the amount of blood pumped per heart beat), and arterio-venous difference in oxygen content (*C*_a_O_2_−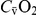) [14]:
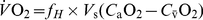
(1)Using *f*_H_ to estimate variation in 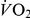 is contingent on the assumption that the oxygen pulse (OP, *i.e.*, the amount of oxygen that is consumed by the animal during a single heart beat) defined as 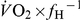, is either constant, or changes in a systematic way [8], [13]. Measurement of *f*_H_ can therefore serve as a useful estimate for MR in birds only once the relationship between *f*_H_ and energy expenditure is established [13], [15], [16]. When this relationship is known, the method may be at least as accurate as the DLW technique [8], [17], while avoiding some of its drawbacks, including the need to recapture animals [13]. Moreover, unlike DLW measurements, *f*_H_ measurements may allow inferences on the metabolic demands of activities that occur at a fine temporal scale, for example, when a bird responds to changes in wind direction during flight [18].

Although soaring-gliding is energetically cheaper than flapping, for relatively small birds it may come at a potentially high cost in terms of fitness, since the overall flight speed is slower [19] and migration may thereby be prolonged [20]. Using Hedenström's [19] cost of transport model, for example, a 5 kg bird is theoretically expected to migrate twice as fast when soaring and gliding than when flapping. In contrast, Hedenström's [19] model predicts that soaring-gliding birds weighing 0.50 and 0.05 kg migrate 1.5 and 3.5 times more slowly, respectively, than when using flapping flight. Nevertheless, a number of small species, such as swifts, swallows, and bee-eaters often soar and glide [e.g., 21], including during migratory flight, suggesting that the assumptions regarding their cost of transport need to be empirically reevaluated.

**Figure 1:**
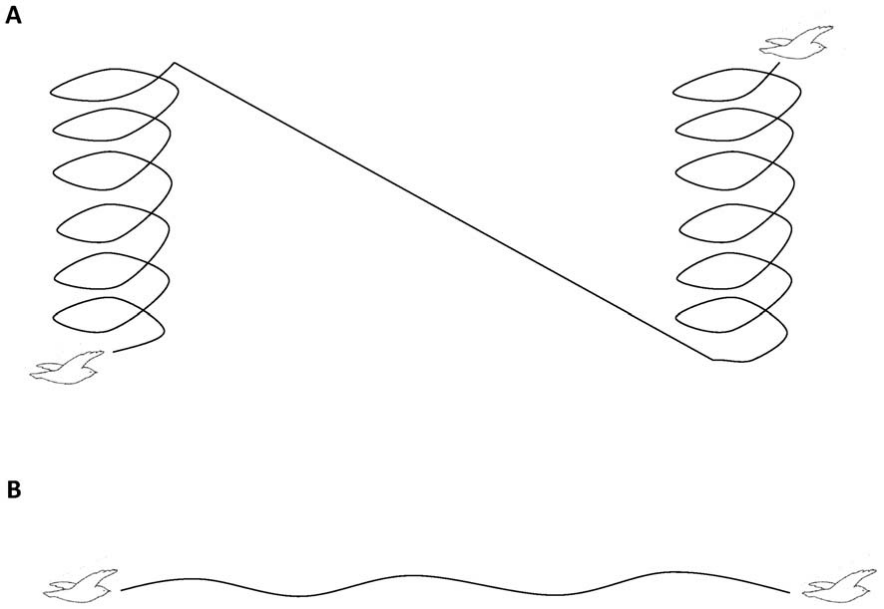
Sketch of European bee-eater flight modes. (A) Soaring-gliding flight employs updrafts created by the heating of the ground for gaining potential energy during soaring by circling over the rising air. During gliding the bird sinks in the air and progresses horizontally. (B) Flap-glide flight that is composed of sequences of short alternating flapping and gliding phases.

To assess the variables of the cost of transport during soaring and gliding in a small bird species, we established the relationship between *f*_H_ and 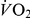 under controlled laboratory conditions and used radio telemetry to measure bird flight mode, flight speed and *f*_H_ in free-ranging European bee-eaters (*Merops apiaster*) during migration, including during rest while staging, and during soaring-gliding and flapping flight (Fig. 1). Theoretical calculations imply that soaring-gliding MR scales with BMR [3] and based on estimates from birds of *m*_b_>0.9 kg [3], [4], [7], [9], we predicted that soaring-gliding MR in bee-eaters is between 1.5 and 3 times their BMR. The data we present bolster the little we know about soaring-gliding MR in birds in general and are the first collected from small migrating birds in the wild.

## Methods

### Field Study

#### Bird trapping and transmitter attachment

Between March and May, in 2005 and 2006, we trapped migrating European bee-eaters at two sites, Eilat (29°34′N 34°58′E) and Grofit (29°56′N 34°04′E), both located in the southern Arava Valley, Israel, on the major eastern Africa - Eurasia migration flyway. The birds were captured with mist-nets near agricultural fields, or in large Rybachy traps [22] at the International Birding and Research Centre in Eilat. Trapped birds were ringed and were individually marked with hair bleach (Blondor Light Powder, Wella Corporation, Woodland Hills, California, USA) applied to the tips of 2–3 of their flight feathers to enable identification in the field [23]. Each bird was fitted with a 1 g transmitter (SP2000-HR, Sparrow Systems, Fisher, Illinois, USA), following Cochran and Wikelski [24]. Transmitters emitted in the 605 MHz band, and the radio signal frequency was modulated by heart and flight muscle electrical potentials [25], [26], enabling the continuous recording of *f*_H_ and wing beat frequency. Due to the high sensitivity of the receiver to changes in signal characteristics, we were able to identify occasions when the birds moved their bodies while perched, for example when preening or engaging in physical interactions with conspecifics.

#### Bird activity and heart beat frequency acquisition and analysis

**Figure 2:**
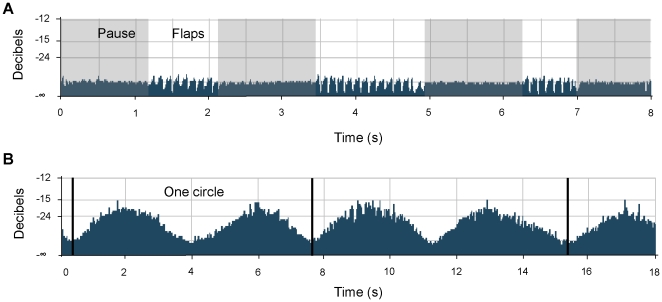
Received audio signals characterizing different flight modes of migratory European bee-eaters during flight. Each activity is characterized by a unique, identifiable signature of the audio power magnitude (decibels) in relation to the baseline power. (A) Flapping flight is characterized by two alternating phases; the first consists of a series of wing beats that are indicated by thick spikes, followed by a pause (grey shaded) of similar duration. (B) Soaring flight is characterized by sinusoidal signal strength due to null reception when the antenna of the tag is facing 180° and 360° towards the receiving antenna while the birds rise in the air in circular path. Thick vertical lines separate between different circles.

**Figure 3:**
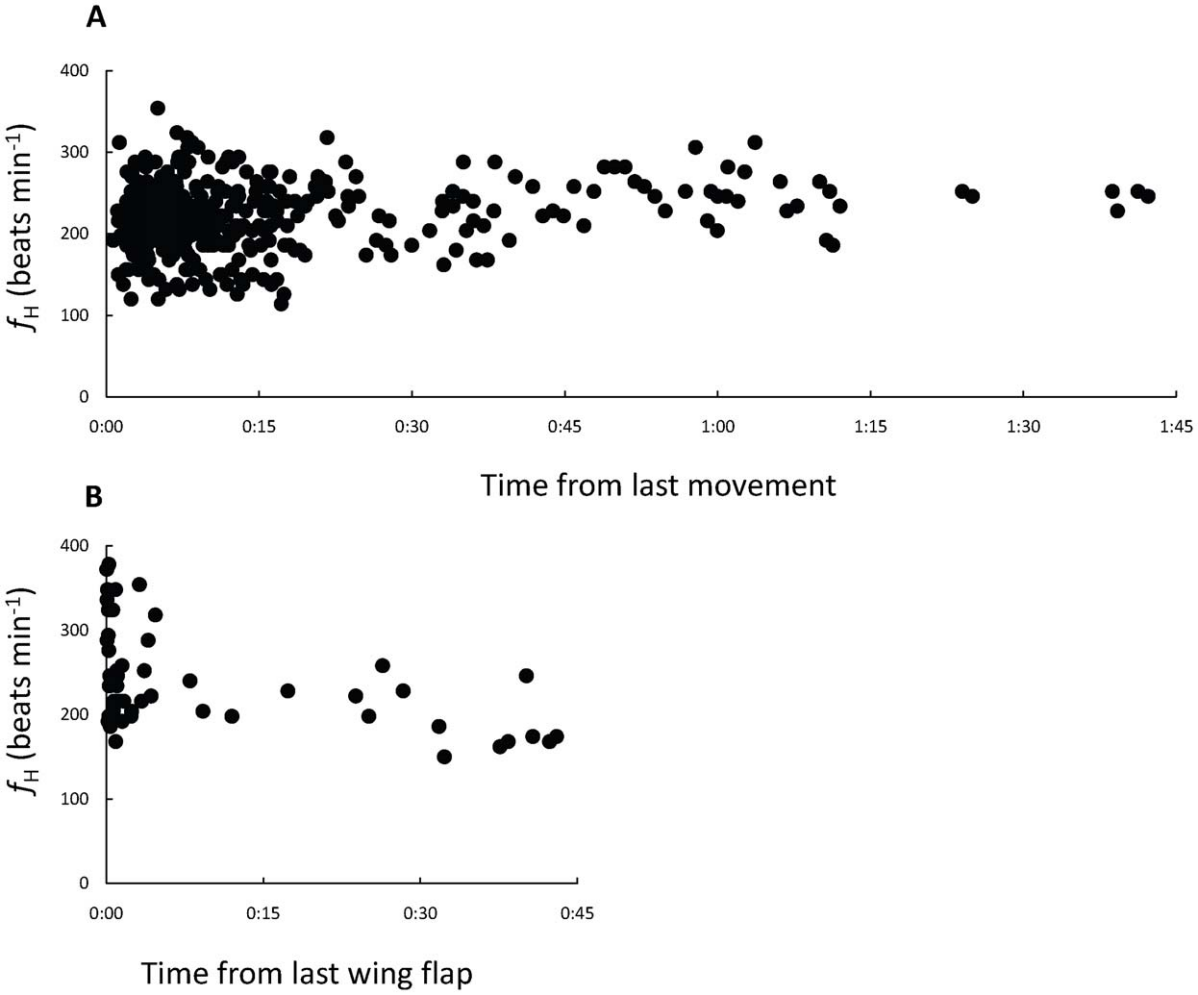
Heart beat frequency (*f*_H_ ) of European bee-eaters in relation to time during activity at rest and during soaring-gliding. (A) *f*_H_ in relation to time from last movement during stopover. (B) *f*_H_ in relation to time from last wing beat during soaring and gliding.

We used two vehicle-mounted telemetry systems, similar to those used in earlier studies [25]–[29], consisting of AR8200 radio receivers (AOR Ltd., Tokyo, Japan) and custom-made Yagi antennae. These were connected to laptop computers running CoolEdit 2000® recording software (Syntrillium Software Corp., Phoenix, Arizona, USA) through digital compressors (αComp, Alto, Italy). During tracking, bird activity was registered by the wing beat signal and the null-reception pattern (see below), permitting us to distinguish between flapping and non-flapping flight (figure 2A). Wing beat and the null reception patterns during different activities were corroborated by occasional visual observations of the individually identifiable tracked birds. Bird movement was followed, and ground speed measured, with the vehicle mounted telemetry system. Recordings from resting birds were scanned and filtered, and we included for analysis only measurements of *f*_H_ in resting birds from which we detected no motion for at least ten minutes (Fig. 3A). In many cases, periods of prolonged rest might have included sleep, but since we have no means to distinguish between resting per se and sleep, we treated all the events during which birds were motionless as prolonged rest.

**Figure 4:**
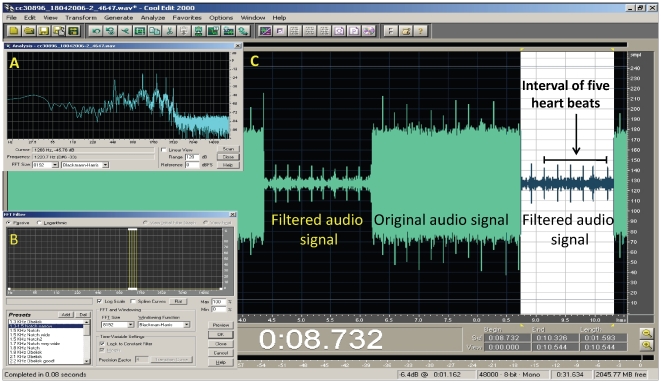
An illustration of heart beat frequency ( *f*_H_) measurement of a European bee-eater using CoolEdit 2000® software. (A) Frequency analysis of the focal audio segment. (B) Application of fast Fourier transform filter according to the frequency analysis of the segment to increase the signal-to-noise ratio. (C) Measuring the time interval of five consecutive heart beats on the computer screen from which *f*_H_ in beats min^−1^ was calculated.

Soaring was characterized by a unique, sinusoidal, decibel level because of null reception when the position of the transmitter's antenna relative to the receiving antenna was at either 180° or 360° to it (figure 2B), a phenomenon observed in earlier telemetry studies of soaring birds [29]. Soaring to gain altitude was typically followed by gliding when the bird flew cross-country. Recordings from soaring-gliding birds were scanned and filtered, and included only measurements of *f*_H_ beginning at least two minutes from the bird's last recorded wing beat (Fig. 3B). We used CoolEdit 2000® software to analyze audio files and applied fast Fourier transform filters to increase the signal-to-noise ratio. We measured *f*_H_ by counting heart beat spikes on the computer screen, averaging five successive inter-beat intervals (Fig. 4), and repeating this procedure every 0.5 min for the whole audio file. When five consecutive spikes could not be counted, for example during very short glides between wing flaps, we counted fewer than five but no less than three consecutive spikes. During flapping we could not distinguish heart beats from wing beats, and therefore *f*_H_ during flapping flight was calculated from the first spikes that followed a series of wing beats during glides. Due to the relatively high *f*_H_ immediately after flapping, sampling five heart beats directly after the last wing beat in a wing beat series took an average of 0.6 s. We assumed that sampling this way only slightly underestimates bird *f*_H_ during actual flapping flight because *f*_H_ subsides only slightly during this short interval.

### Laboratory Study

#### Bird trapping and experimental design

During May 2009 we trapped a total of twelve migrating European bee-eaters at Grofit and transported them to the Jacob Blaustein Institutes for Desert Research at Midreshet Ben-Gurion (30°52′N, 34°47′E). The birds were housed in an aviary (4×2×2.5 m) and were provided with crickets, mealworms and water *ad libitum*. After a habituation period of 24 hours, we equipped each with a radio transmitter as described above, and examined the relationship between their *f*_H_ and MR using indirect calorimetry in metabolic chambers. We monitored their *f*_H_ while simultaneously measuring 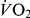 and carbon dioxide production (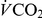). The *m*_b_ of three birds continuously decreased during the first 48 hours following capture; they were released. Two additional birds were released after their radio signals deteriorated and *f*_H_ could not be measured. The signals in three other individuals were viable for only one or two sessions, and we did not include this data in the analysis. Therefore, laboratory analysis included data from four individuals. We usually measured one bird at a time in the gas exchange system, but sometimes, we measured two birds simultaneously, each in a separate chamber.

In order to elicit as wide a range of *f*_H_s as we could without exercising the birds, the ambient temperature (*T*_a_) of the metabolic chambers was varied to include *T*_a_s of 10°C, 17.5°C, 25°C, 32.5°C, and 40°C. This range is similar to the natural range of air temperatures (measured in Eilat during spring 2006 by the Israeli Meteorological Service: 12 to 41°C, average 26.5°C), that these birds experienced during their spring migration in the study area. Measurements on each individual began at least 20 min after it was placed in the metabolic chamber, after habituation to the situation, as judged by the 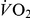 trace leveling off. In some of the sessions, determined at random, we started the experiment when the *T*_a_ in the chamber was 10°C and, after measuring 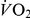 at this *T*_a_ for 10 min, we increased the temperature to 17.5°C, waited 20 min, assessed whether the 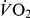 trace had leveled off, measured 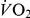 again for 10 min, and then increased *T*_a_ in similar fashion until measurements at all five *T*_a_s were made. In other sessions, we started the experiment when the *T*_a_ in the chamber was 40°C and used a decreasing *T*_a_ sequence. 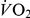 was measured during both photophase and scotophase to encompass both resting and sleeping states.

#### Quantifying the relationship between heart beat frequency and metabolic rate

Air from outside the building was pumped via a purge gas generator (PCDA-1-12-m-32-C, Pure Gas, Broomfield, Colorado, USA) that removed CO_2_ and water vapor to less than 1 ppm through metabolic chambers where birds were placed. The volume of each metabolic chamber was 800 ml, but was functionally reduced to about 750 ml by the bird's volume. The average flow rate was 600 ml min^−1^, meaning that the air in the chamber was turned over in about 75 seconds. Gases were continuously measured using an infrared CO_2_ analyzer and an O_2_ analyzer (models CD-3A and S-3A, respectively; AEI Technologies, Naperville, IL, USA) coupled to a programmable multiplexer (Sable Systems International, Las Vegas, NV, USA). 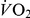 and 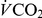 were calculated as ml gas min^−1^ using equations 2 and 3:
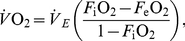
(2)and
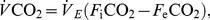
(3)where 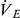 is the mass flow of gas exiting the metabolic chamber in ml min^−1^, *F*_i_O_2_ and *F*_i_CO_2_ represent the fractional concentrations of O_2_ and CO_2_ entering the metabolic chambers, and *F*_e_O_2_ and *F*_e_CO_2_ represent the fractional concentrations of O_2_ and CO_2_ exiting the metabolic chambers [30]. The average (± SD) respiratory exchange ratio (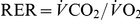) for bee-eaters was 0.75 (±0.20). We assumed that mean RER for fat is 0.71 and for protein in birds is 0.74 [31], and converted 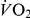 (ml O_2_ min^−1^) to units of power (W), further assuming that the birds metabolized a combination of 90% fat and 10% protein [32]. For each ml of O_2_ consumed during protein catabolism, 18.70 J are released, while for lipid, 19.8 J are released [31]. Therefore, we assumed that the birds released 19.69 J for each ml of O_2_ consumed. Hence, by converting 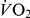 (ml O_2_ min^−1^) to power (watts = J s^−1^), we estimate that the uptake of 1 ml O_2_ s^−1^ equals a power input of 0.33 W. We used the minimum value of 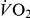 of each bird measured during scotophase, considered the mean of these 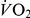 values as basal 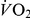, and used the RER as specified above to estimate BMR. We examined the effects of *T*_a_ on 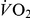 and *f*_H_, but since our measurements were made in a certain, limited, range of *T*_a_s, no objective method (e.g., [33]) was applied to estimate the thermal neutral zone of the birds.

### Statistical Analysis

We compared wing beat frequency during foraging flapping flights and cross-country flapping flights by independent sample t-test with unequal variances, following a Levene's test for equality of variance (p<0.001). We applied an independent sample t-test with equal variances, following a Levene's test for equality of variance (p = 0.47) to compare bird ground speed during cross-country soaring-gliding and flapping flight. We used the Wilcoxon paired signed rank test using exact probability calculations [34] to compare mean *f*_H_ from the field between pairs of activities of each individual. For example, we compared the average resting *f*_H_ of each individual measured during stopover with its own average *f*_H_ measured during migratory cross-country soaring-gliding flight.

**Figure 5:**
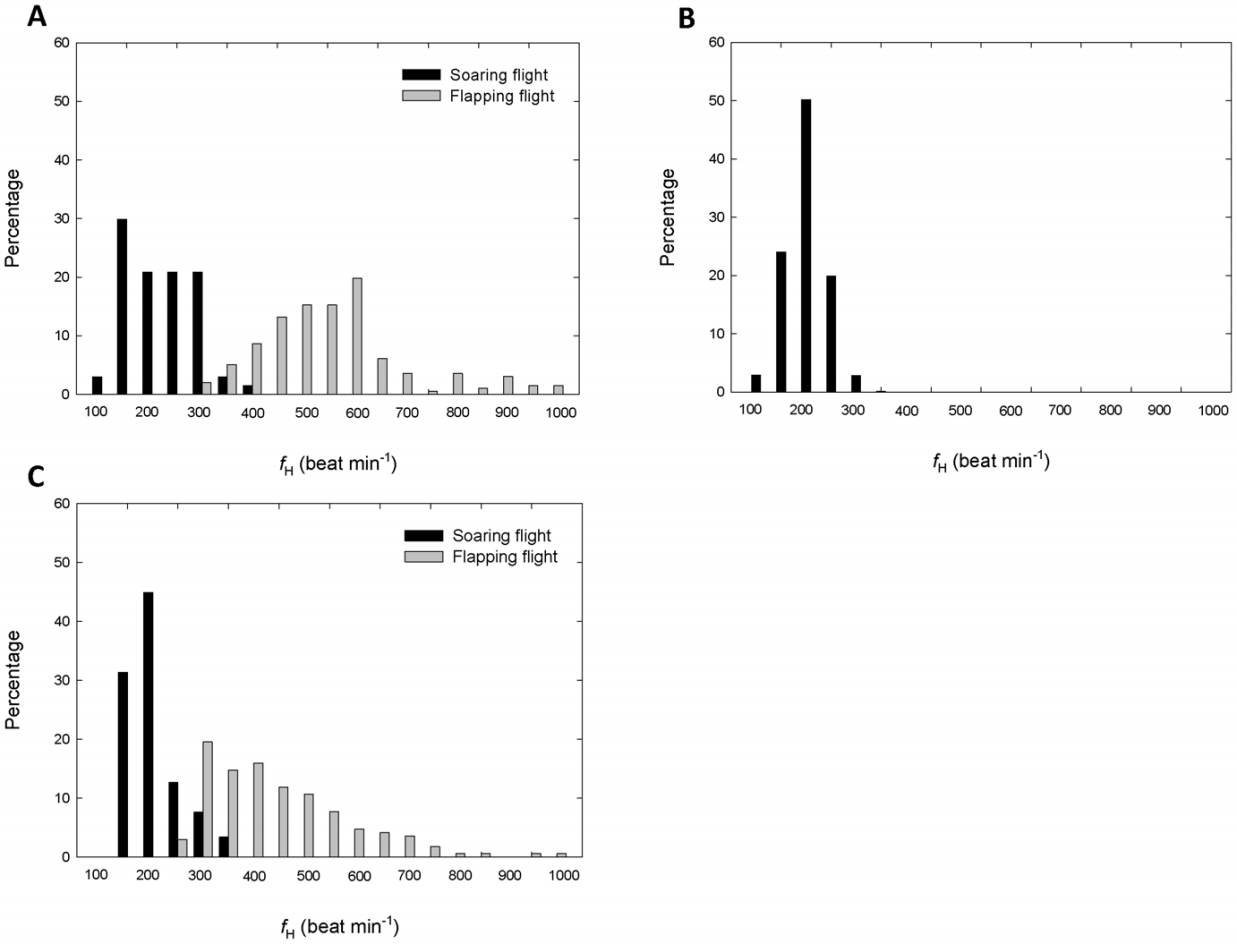
Distribution of heart beat frequency ( *f*_H_) of European bee-eaters during different activities and migration stages. (A) Soaring and flapping flights during stopover. (B) Rest during stopover. (C) Soaring and flapping during cross-country flight.

We used ANCOVA to test for the effect of the individual (independent categorical random factor) and the period of the experiment (photophase or scotophase, independent categorical fixed factor) on the relationship between *f*_H_ (independent factor) and 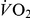 (dependent factor). Initially we considered all data from the experiment; however because we had different numbers of data points from each individual, we weighted the number of observations so that each bird was equally represented [35], [36]. Following this analysis, we did a major axis type-II regression to establish the relationship between *f*_H_ (independent factor) and 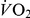 (dependent factor) using a Matlab® code provided by Peltzer [37]. Since resting and soaring *f*_H_ during stopover, as well as soaring-gliding *f*_H_ during cross country flight, rarely exceeded 350 beats min^−1^ (Fig. 5), we excluded from this analysis *f*_H_ values greater than 350 beats min^−1^. We first ran the regression using all data points from the experiment that met this criterion (i.e., <350 beats min^−1^) and then repeated the analysis using equal number of data points from each individual, and we used a random number generator (in Matlab®) to remove measurements from those birds in which an excess were made. The latter analysis was done in order to reduce potential bias due to the unequal contribution of data from different individuals to the overall dataset because we could not used weighted data in this particular statistical procedure. We also applied major axis type-II regression to test if OP (oxygen pulse; dependent factor) changes systematically with 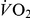 (independent factor), using linear and log models. To test the effects of *T*_a_ on 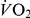 and *f*_H_ we used one-way ANOVA followed by Bonferroni post-hoc test, and ran separate analyses for photophase and scotophase.

We calculated the standard error of the estimate (SEE) of 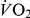 for resting and soaring-gliding during stopover and for soaring-gliding during cross-country migratory flight, based on the formula of Green et al. [38]:
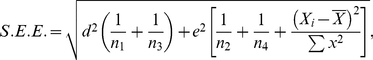
(4)where *d* ^2^ is the error associated with the variation between individuals during the laboratory experiment, *n*_1_ is the number of bee-eaters studied in the laboratory, *n*_3_ is the number of bee-eaters whose *f*_H_ was measured in the field, *e* ^2^ is the error associated with the scatter around the regression line, *n*_2_ is the number of data points in the regression, *n*_4_ is the number of data points used for calculating the average *f*_H_ from the field, 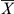 is the average value of *f*_H_ used in the regression, *X_i_* is the average value of *f*_H_ from the field from which σ_1_ is to be estimated and 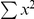 is the sum of all the squared values of *f*_H_ used in the regression. To estimate the average and standard deviation of the power input associated with estimated average 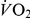 from the field, we used RER as specified above. All values are reported as means ±1 standard deviation, unless otherwise indicated.

### Ethics Statement

The tips of several flight feathers of the tracked birds were marked by making them sandy colored using human hair bleach that was applied for 10 min [23]. After applying the lightener we washed the remains from the feathers with water and left the feathers to dry. The birds were anesthetized with a mixture of Isoflurane and air during the attachment of the radio tags following Cochran and Wikelski [24], and we minimized bird handling time by releasing the birds as soon as possible after completing the ringing and transmitter attachment procedures, always within an hour of trapping. Tag mass was 2.05% of the *m*_b_ of the bird with the lowest *m*_b_ (48.8 g), and 1.78% of the average *m*_b_ of all the birds (56.3 g). Birds were released in the field near conspecifics, and we followed them after release by radio tracking and with binoculars. We did not detect any abnormal behavior of the birds during different activities such as foraging flights, rest, and later on during migratory flights that we compared to untagged conspecifics around them. For example, during migratory flights tagged birds were found inside migrating flocks and did not show signs of lingering; we could see no difference in their flight compared to untagged birds. The same tags were applied to ∼17 g spotted antbirds (*Hylophylax naevioides*) whose *m*_b_ is about one third that of the bee-eaters, and no adverse effects of the tag were detected [39]. Raim [40] found that the same tags fell off the backs of 60 brown-headed cowbirds (*Molothrus ater*) in 10–14 days, with an upper limit of 24 days. Bird trapping permits were obtained from the Israel Nature and Parks Authority (permits 2005/22055, 2006/25555) and the experimental procedure was approved by the Animal Care and Use Committee of the Hebrew University of Jerusalem (permits NS–06–07–2 and NS–09–11652–4).

## Results

**Figure 6:**
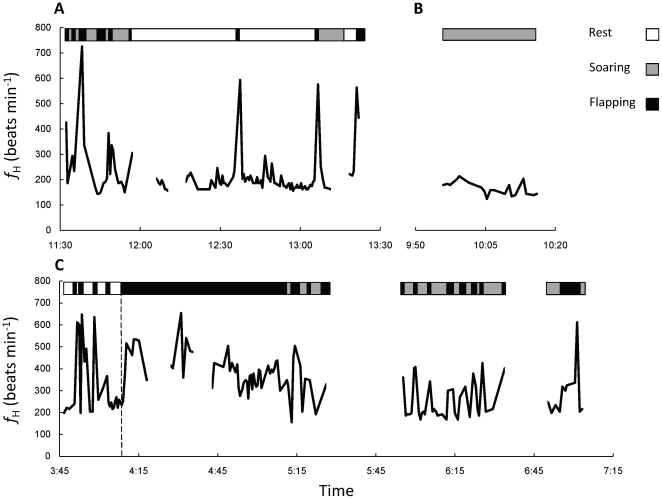
Heart beat frequency (*f*_H_) traces of two European bee-eaters engaged in different activities during stopover and cross-country flight. (A) *f*_H_ of bird no. C48417 during stopover on May 5 2006 in relation to bird activity. A sample of this data can be heard in Audio S2. (B) *f*_H_ of the same bird on the following day, 6 May 2006, during cross-country flight. A sample of this data can be heard in Audio S1. (C) *f*_H_ of bird no. CC30955 on May 25 2006. The bird was followed during stopover in the early morning and then took off (dashed vertical line) for cross-country flight in which it was followed for a distance of 90 km from the stopover site until its signal was lost. Time is GMT; add two hours for local time.

During the spring seasons of 2005 and 2006, we followed 34 migrating bee-eaters in the southern Arava Valley, Israel. After the birds stopped over in the area for one to nine days, we were able to track 11 of them during cross-country migratory flights of up to 230 km. All the bee-eaters took off for migratory flight during the day, and their *f*_H_ was recorded using the two vehicle-mounted telemetry systems until their signals were lost. Following field observations, we distinguished between two modes of flight used by the birds during cross-country flight: (1) flapping flight, characterized by a series of rapid wing beats lasting an average of 1.39±1.84 s (N = 40 series from six individuals), separated by 1.28±1.19 (N = 40 series from six individuals) second-long pauses (Fig. 2A), and (2) sustained soaring-gliding flight (Fig. 2B and audio S1), followed by gliding without flapping (9.5±14.2 minutes from last wing beat, range 2.0–43.0 minutes, N = 53 from six individuals). Flapping flight in European bee-eaters thus consists of flap-glide flights with a power fraction (the proportion of the cycle during which the bird actively flaps) of 0.5 (see [41], [42], and compare with [43]). During foraging at a stopover, we again distinguished flapping flight from soaring flight, when the birds circled and rose above the stopover sites without making any horizontal progress by gliding. In addition, we defined prolonged motionless rest during stopovers (33.5±53.9 minutes from last body motion, range: 10.0–278.3 minutes, N = 385 from nine individuals; audio S2). In Figure 6 we present raw traces of *f*_H_ and corresponding activity from two birds tracked during stopover and cross-country flight, and figure 5 shows the distribution of *f*_H_ during stopover for soaring flight, flapping flight and rest, as well as during cross-country flight for soaring-gliding and flapping flight.

**Figure 7:**
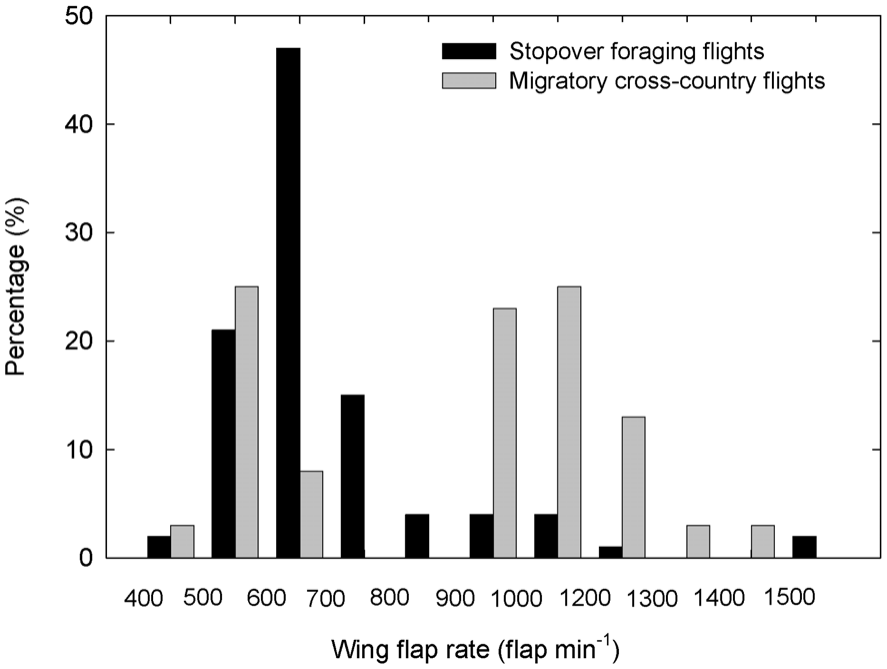
The distribution of wing beat frequency of European bee-eaters during stopover (foraging flights) and cross-country flight (flap-glide flights).

Wing beat frequency during stopover feeding flights (696.9±180.6 min^−1^, N = 112 from nine individuals) was significantly lower (independent sample t-test, df = 54.5, t = 4.1, p<0.001) than wing beat frequency during migratory cross-country flapping flight (874.8±247.6 min^−1^, N = 40 from six individuals). The distribution of wing beat frequency during stopover and cross-country flight is presented in figure 7. Ground speed during flapping flight, averaged for each individual, was 10.1±6.5 m s^−1^ (N = 7 birds), and was 10.3±1.5 m s^−1^ (N = 5) during soaring-gliding. Generally, flapping flight took place into headwinds (2.8±4.9 m s^−1^), while soaring-gliding took place under variable wind conditions (0.3 m±3.0 m s^−1^; [44]). Mean bird air speed during flapping flight was 12.9±5 m s^−1^, not significantly different from mean air speed during soaring-gliding (10.6±3.2 m s^−1^; independent sample t-test, df = 10, t = 0.92, p = 0.38).

**Figure 8:**
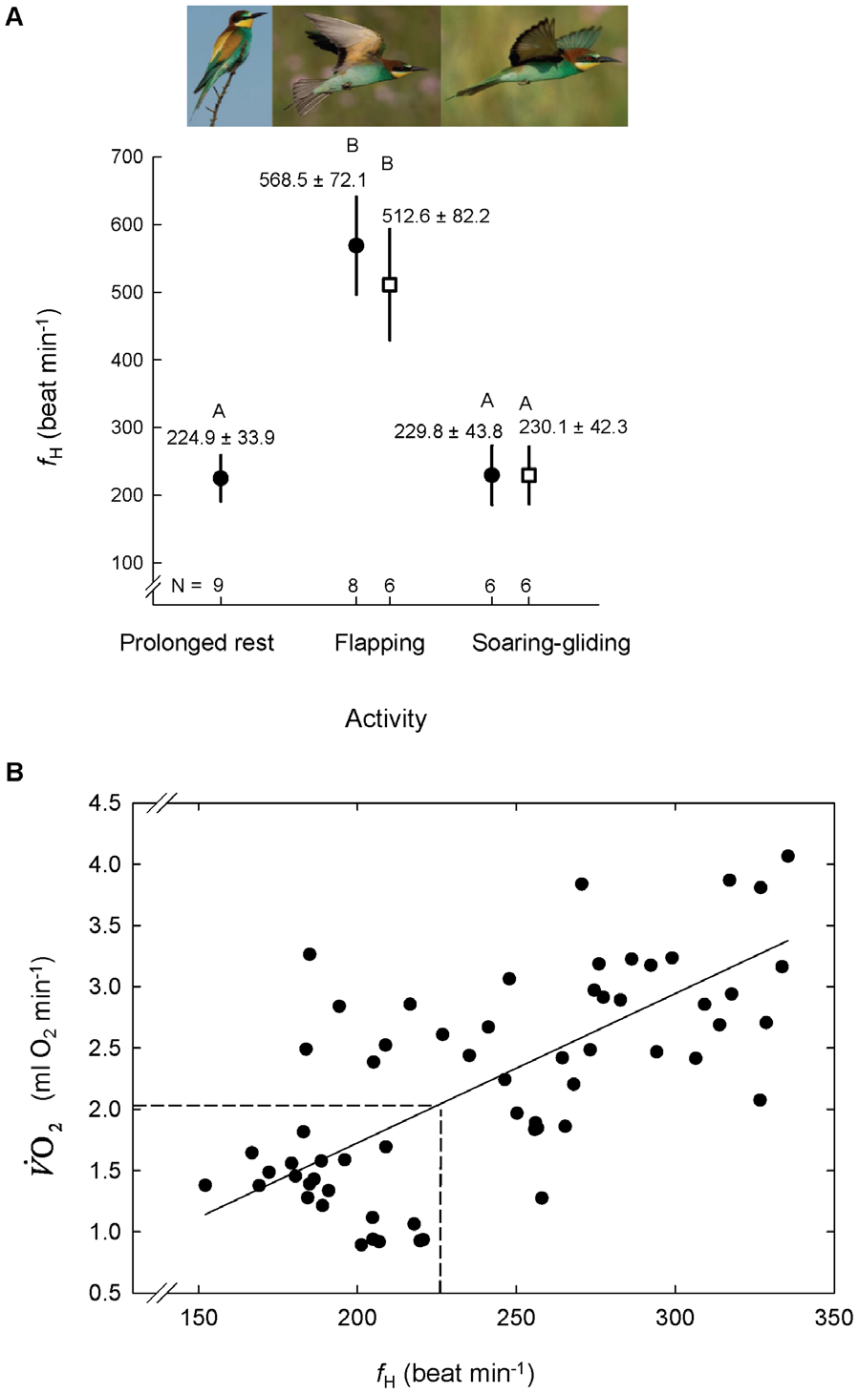
(A) Heart beat frequency ( *f*_H_) of European bee-eaters measured in the field in relation to bird behavior, and (B) Laboratory 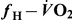 relationship. In panel A the symbols represent mean ± standard deviation *f*_H_ of bee-eaters resting for prolonged duration and in different flight modes recorded during stopovers (filled circles) and migratory cross-country flights (open squares). Different letters above the bars indicate groups that differed statistically. N = number of birds whose *f*_H_ was recorded during each activity. The photographs above the figure depict each activity (photo credits: Daniele Occhiato). In panel B the trend line is illustrated following a major axis model II regression with N = 63; see Table 2 for regression statistics. Dashed vertical line indicates the average soaring-gliding *f*_H_ recorded in the field, and the dashed horizontal line depicts the corresponding 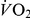 value.

Heart beat frequency of bee-eaters measured during cross-country soaring-gliding flight or during stopover soaring flight was statistically indistinguishable from *f*_H_ measured during prolonged (>10 minutes), motionless, rest (Wilcoxon paired signed rank test, N = 6 birds, Z = 0.73, p = 0.46 and N = 6, Z = 0.11, p = 0.92, for cross-country flights and stopovers, respectively). Average *f*_H_ during cross-country soaring-gliding flight was not different from that measured during soaring-gliding flight while the birds were stopping over (N = 4, Z = 0.4, p = 0.71). In addition, *f*_H_ during flapping foraging flight in stopover was statistically indistinguishable (N = 5, Z = 1.5, p = 0.14) from *f*_H_ during cross country flapping flight. Heart beat frequency during flapping flight was 2.2 to 2.5 times higher than that during soaring, gliding or resting. These differences were statistically significant in all paired comparisons; for example flapping vs. soaring-gliding flights during stopover (N = 8, Z = 2.5, p = 0.012) and cross-country flight (N = 6, Z = 2.2, p = 0.028). Figure 8A includes the average, per bird, *f*_H_±SD.

We found that individual birds had no significant effect on the relationship between *f*_H_ and 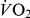 when tested using both un-weighted and weighted data. The time in which the measurements took place was a significant factor, with 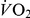 during scotophase being significantly lower than 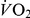 during photophase. 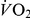 covaried significantly with *f*_H_ (Table 1). The results of major axis type-II regression applied to establish the relationship between *f*_H_ (independent factor) and 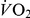 (dependent factor) were similar in the two models that used either all data points or equal numbers of data points from each bird (Table 2). Figure 8B shows the relationship between *f*_H_ and 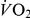 using all the data from the birds. To estimate the standard error of estimated 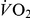, we applied equation 3 [38], using both laboratory and field data. Table 3 shows the average and SEE of estimated 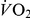 of European bee-eaters during prolonged resting, stopover soaring flights, and cross-country soaring-gliding flight using the two different regression equations from table 2. Estimated 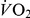 was higher by 0.3–1.4% by the regression equation that used all data points than by the regression that used equal number of data points from each bird, and SEEs of estimated 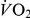 were 33.6–54.7% lower in the former regression than in the latter. Assuming that 19.69 J were released for each ml of O_2_ consumed by the birds, and based on the regression that included all data points, we estimated MR during cross-country soaring-gliding flight to be 0.691±0.312 W.

**Table 1:** ANCOVA results^1^^,^ 2 from analysis of the effects of individual birds (Bird ID, random categorical factor) and the period of the measurements (day/night, fixed categorical factor) on the relationship between *f*_H_ (covariate) and 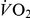 in European bee-eaters.

	Data not weighted					Data weighted				
Source	df	Sum of squares	Mean square	F	p	df	Sum of squares	Mean square	F	p
Intercept	1 (48.59)	0.05 (20.46)	0.05 (0.42)	0.117	0.73	1 (64.85)	0.005 (27.40)	0.005 (0.42)	0.12	0.91
*f*_H_	1 (69)	47.68 (26.48)	47.68 (0.38)	124.3	<0.001	1 (101)	60.61 (39.35)	60.61 (0.39)	155.56	<0.001
Period	1 (8.00)	4.26 (1.98)	4.26 (0.25)	17.2	0.003	1 (3.55)	8.95 (1.83)	8.95 (0.52)	17.37	0.018
Bird ID	3 (3.03)	2.27 (0.61)	0.76 (0.20)	3.7	0.153	3 (3.07)	2.30 (1.62)	0.77 (0.53)	1.45	0.38
Period×Bird ID	3 (69)	0.61 (26.48)	0.20 (0.38)	0.5	0.67	1 (101)	1.59 (39.35)	0.53 (0.39)	1.36	0.26

1 Error terms of each factor in the statistical model appear in parentheses.

2 Data was weighted according to the inverse of the proportion of an individual bird's samples size in the sample size from all birds.

**Table 2:** Major axis type 2 regressions analysis^1^ results from analysis of the association between the independent factor *f*_H_ and the dependent factor 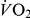 in European bee-eaters.

	All data points	Equal number of data points from each bird
N	63	24
Slope (±SD)	0.0122 (±0.0017)	0.0085 (±0.0026)
p Slope	<0.001	0.005
Intercept (±SD)	−0.7135 (±0.4097)	0.1113 (±0.6383)
p Intercept	0.092	0.87
R^2^	0.46	0.31
Overall p	<0.001	0.005

1 Excluding *f*_H_>350 beat min^−1^.

**Table 3:** Estimated (average ± SEE) 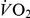 of European bee-eaters during prolonged resting, stopover soaring, and cross-country soaring-gliding flight.

Activity / type of regression	All data points	Equal number of data points from each bird
Prolonged resting	2.030±1.349	2.023±2.087
Stopover soaring flight	2.090±0.968	2.065±1.309
Cross-country soaring-gliding flight	2.093±0.944	2.067±1.261

**Figure 9:**
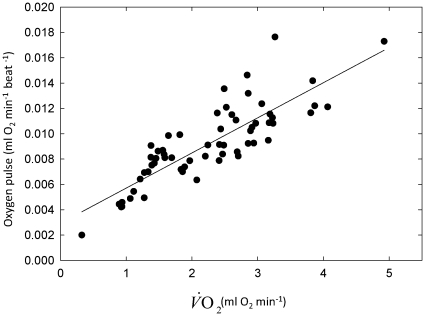
The relationship between 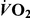 and oxygen pulse (OP) in European bee-eaters for *f*_H_ values<350 beat min^−1^.

In both regressions the slope 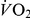 vs. *f*_H_ was significantly different from zero while the intercept was not (Table 2). This implies that, for the range of *f*_H_ examined in the present study (*f*_H_<350 beats min^−1^), the OP is constant or changes in a systematic way with *f*_H_ [13]. We specifically tested for this effect by applying major axis type-II regression with the dependent factor OP and the independent factor 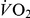 [13]. We found that the relationship was best described by a linear regression (Fig. 9), for which the equation is:
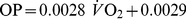
(5)(N = 63 from four birds, R^2^ = 0.716, and P<0.001), indicating that *f*_H_ can predict 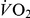 in this range of *f*_H_ with reasonable accuracy [13].

**Figure 10:**
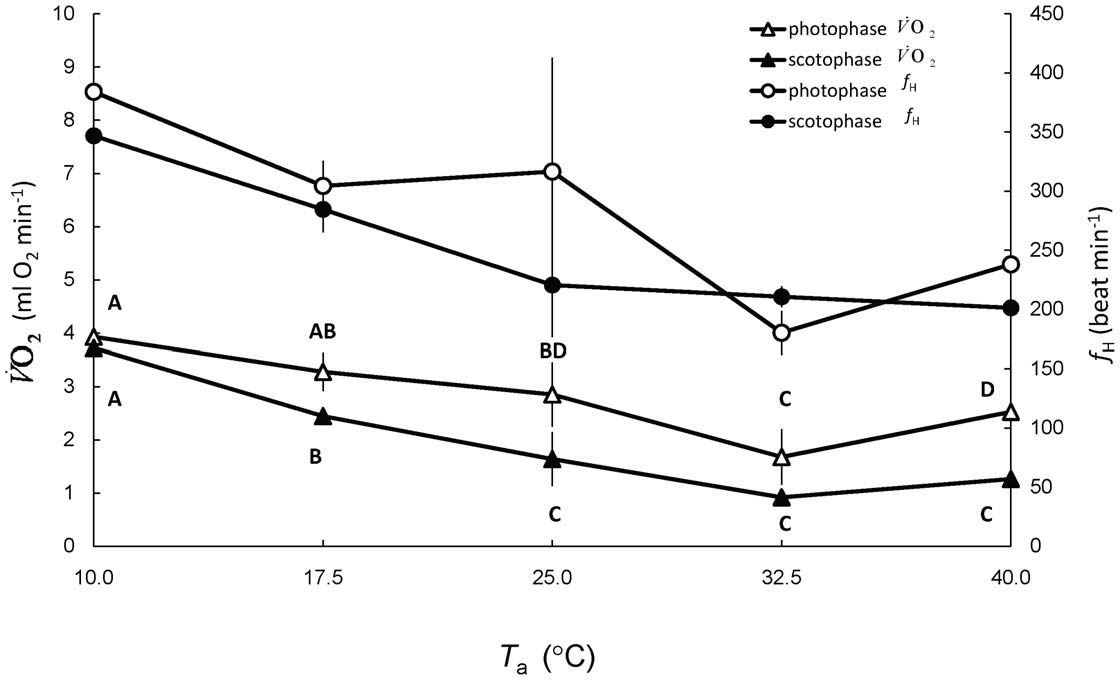
Variation in 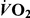 and *f*_H_ in relation to ambient temperature (*T*_a_) and period of measurement. Different letters above 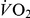 data for photophase and below it for scotophase indicate significantly statistical difference (p<0.05) between groups.

We found that bee-eater 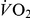 during scotophase was significantly related to *T*_a_ (Fig. 10; one-way ANOVA, F_4,34_ = 38.84, p<0.001). 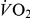 was not statistically different when birds were exposed to *T*_a_s of 25°C, 32.5°C, and 40°C (Bonferroni post-hoc test; p>0.106), while it was significantly higher when *T*_a_s were lower than 25°C (p<0.006). Bird 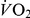 during photophase was also significantly affected by *T*_a_ (Fig. 10; F_4,42_ = 21.30, p<0.001). 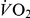 was significantly lower at 32.5°C than at any other *T*_a_ (Bonferroni post-hoc test p<0.018).

To estimate BMR we used the minimum 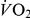 of each bird, but we did not include an extremely, and unrealistically, low minimum 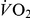 value recorded from one of the birds (0.33 ml O_2_ min^−1^; Dixon's outliers test, p<0.1). Instead we used the second to minimum value from that bird for the calculation. Basal 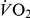 was estimated to be 1.09 (±0.16) ml O_2_ min^−1^. Bird *f*_H_ during minimal 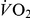 measurements was 220.4 (±26.0) beat min^−1^, and we estimated the BMR of European bee-eaters to be 0.360 (±0.053) W. This estimate is ∼30% lower than the value predicted by McKechnie and Wolf's [29] equation relating BMR to *m*_b_ in birds (0.51 W). We estimate that MR during cross-country soaring-gliding flight in European bee-eaters (0.691±0.312 W) was 1.92 times their BMR.

## Discussion

The *f*_H_ in soaring-gliding European bee-eaters in relation to resting *f*_H_ is, to our knowledge, the lowest recorded thus far among soaring-gliding birds and certainly the first in free-flying small birds. Previous investigations of birds of 0.9 to 10 kg [4], [7], [9], [11], [12] found that soaring or gliding *f*_H_ was 1.3–2.0 times resting *f*_H_. Our estimate for MR of migrating European bee-eaters during cross-country soaring-gliding flight, stopover soaring flight, and prolonged rest were about twice their BMR. This estimate is similar to that reported by Bevan et al. [9] for breeding black-browed albatrosses during dynamic soaring. With the exception of the study of Bevan et al. [9], our estimated soaring-gliding MR in relation to BMR, is 0.67 to 0.83 the value found for other species. It is possible that different methodologies are responsible for these differences. In the study of Baudinette and Schmidt-Nielsen [4], it is possible that the conditions of the wind tunnel, namely the small test section that was only slightly wider than the birds' wingspan, and the relatively turbulent flow of air, resulted in elevated MR during gliding, and during the measurements the birds often extend their feet to land (B. Pinshow, *personal observations*). The estimation of soaring MR reported by Adams et al. in wandering albatrosses [7] was based on a combination of DLW measurements and bird time-activity budgets. The latter were measured in different albatross populations and may therefore not reflect the actual budgets of the DLW studied birds (see details in [7]).

Measurement of *f*_H_ can serve as a useful estimate for MR in birds [9], [15], [16], [17], but only once the relationship between *f*_H_ and 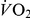 has been established [8], [13], [15], [16]. Although OP changed systematically with 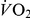 in resting bee-eaters in the laboratory (Fig. 9), this relationship may not be general to all birds and during different activities. Since instantaneous 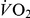 cannot be measured in the field in free ranging birds, or other animals, to estimate field MR from *f*_H_, one must assume that the *f*_H_ - 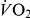 relationship established in the laboratory holds true for birds in the wild. This may well be the case in soaring-gliding flight when the birds do not flap their wings, or in other organisms that use power to statically support a weight against gravity. In the study of Bevan et al. [8], [9], the 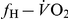 relationship was based on measurements from birds that did not support weight in flight, but rather walked on a treadmill and thus were exercising their leg muscles. Maas et al. [46] reported that *f*_H_ and 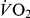 were correlated, and that 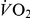 was only slightly higher in humans supporting a static load, with forearms horizontal while holding water-containing Jerrycans weighing 4–10 kg for 10 minutes, than when at rest. When static load support was combined with walking on a treadmill, the 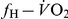 relationship increased dramatically, probably because OP changed between these two activities (see also [15]). The 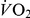 estimates obtained using measurements of treadmill walking or exposure to variable *T*_a_ must therefore be re-evaluated in the future using other methodologies that are not burdened by the assumption regarding the 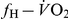 laboratory relationship. This may be achieved, for example, using modern, tiltable, wind tunnels (e.g., [47]) where birds can glide continuously [48].

Our finding that bee-eaters may have low soaring-gliding MR, and that soaring-gliding cross-country flight does not incur a penalty of slower progress on the journey provides a plausible explanation for why several species of small birds soar and glide during migration. Consequently, we suggest that assumptions regarding the cost of transport in birds using different flight modes (e.g., [19]) should be re-evaluated. Low cost of transport may imply low energetic demands of major activities in their life cycle, associated not only with long-distance migration, but also with foraging, for example, during which birds were frequently seen gliding (usually just after prey capture, N. Sapir *personal observations*).

Sustained soaring-gliding flight can only be used under particular meteorological conditions [3], and its use may be limited by headwinds along migratory flyways [19]. Hedenström [19] proposed that small, soaring-gliding, migratory species are particularly sensitive to headwinds due to their low flight speed. Since soaring-gliding was used under variable wind conditions, including headwinds, this factor is probably of minor importance. An additional factor, atmospheric convection, was found to limit soaring-gliding in bee-eaters [44]. Therefore, unlike flapping flight, soaring-gliding flight in bee-eaters, and probably in other avian species, might be largely constrained in time and space by occurrence of the necessary meteorological conditions.

We conclude that the combination of low MR and relatively high speed of progression during soaring-gliding in migrating European bee-eaters may explain the propensity of small bird species to soar and glide because the cost of transport is lower than that of flapping, although this mode of flight is limited to when conditions are appropriate. Our findings and those of Bevan et al. [9] also call for reconsideration of the energy cost of soaring-gliding in birds that is commonly assumed to be 3–4 times BMR (for example in [19]). Finally, our data support Pennycuick's [3] theoretical conclusion that soaring-gliding MR scales with *m*_b_ with a similar scaling factor to that of BMR.

## Supporting Information

Audio S1 Soaring flight heart beat recording. The file contains 24s of heart beat recording of bird C48417 during sustained soaring within a migratory cross-country flight over southern Israel on 6 May 2006. Heart beat frequency varies within this section between 161.0 to 196.7 beats per minute. Bird circling rate during soaring is 9.6s per circle (completed 2.5 circles in 24s).
(1.15 MB WAV)

Audio S2 Prolonged rest heart beat recording. The file contains 24s of heart beat recording of bird C48417 during prolonged rest while stopping-over in southern Israel on 5 May 2006. Heart beat frequency varies within this section between 170.0 to 180.3 beats per minute.
(1.15 MB WAV)
